# Supplementary material for: Diverse modulatory effects of bibenzyls from Dendrobium species on human immune cell responses under inflammatory conditions
Source: PLoS One. 2024 Feb 1;19(2):e0292366. doi: 10.1371/journal.pone.0292366 (PMC10833532; doi:10.1371/journal.pone.0292366)
Supplement: Attachment — Submitted filename: Comments to the authors_PONE.docx [file pone.0292366.s002.docx]

Comments to the authors:

The manuscript entitled “Immunomodulatory effects of bibenzyls from Dendrobium species on diverse human immune cell types under inflammatory conditions” is interesting as it explores the potential of natural compounds in immunomodulation under inflammatory conditions.

The authors have put in good effort in the study, but the manuscript is not well written and there are many grammatical mistakes. Also, the flow and explanation of findings are not up to the mark.

There are multiple queries that are needed to be addressed:

1. In the abstract “Our study demonstrated board immunomodulatory effects…” correct broad. Also change the sentence to: Our study demonstrated broad immunomodulatory effects of Dendrobium compounds on multiple immune cell types including CD14+ monocytes.
2. In the abstract “This broad spectrum of activity on immune responses of Dendrobium compounds may lead to effectively therapeutic potential of these compounds in complex disease conditions including inflammation.” Replace effectively with effective. Also, this sentence is an exaggeration of the findings and hence can simply be written as “The broad spectrum of activity on immune responses of Dendrobium compounds may have promising therapeutic potential in various diseases including inflammation”.
3. The above 2 points are just an example of the grammatical errors and mistakes cited only from the abstract. The authors are recommended to get the whole manuscript checked by a writing expert.
4. Lines 48-51 can be put in the discussion section.
5. There are many sentences in the introduction section which have grammatical mistakes and thus change the context and meaning.
6. Line 86-87 says that LPS treatment is decreasing the TNF expression while from the results LPS is increasing the expression and the treatment with bibenzyls are reducing the expression of TNF.
7. What’s the rationale for taking 4 different Dendrobium species for isolating bibenzyls?
8. There is no information about the blood donor's age, gender, and health conditions from which PBMCs were isolated.
9. From Figure 3, it can be observed that treatment with compound 2 decreases the TNF in CD14^+^ monocytes even at 1µM concentration and is dose-dependent as well till 10 µM condition. Why didn’t the authors consider this compound for further studies when less concentration is showing significant results?
10. In Figure Legend 3, the authors should clearly state the treatment conditions. The current statement is confusing and misleading.
11. What’s the rationale for taking CD45^+^ cells for the cytotoxicity experiment? Why not the CD14^+^ monocytes taken for the study when the previous experiment shows the effect on these cells?
12. For deep immune profiling experiments, authors should consider PBMCs from more healthy donors. It is observed that the basal level of active immune cells of one of the donors is high without any stimulation.
13. Authors should consider isolating PBMCs from individuals with inflammatory conditions and then treating them with these compounds to substantiate their findings with more results.
